# Exploring demographic and organisational variations in patient safety culture: a cross-sectional, multicentre study in operating theatres of six Norwegian hospitals

**DOI:** 10.1186/s12913-026-14460-y

**Published:** 2026-03-31

**Authors:** Niko Aleksander Skaar, Ann-Chatrin Linqvist Leonardsen, Kristin Sandal Berg, Tonje Bjørndal Braaten, Maria Strandås, Susanne Sørensen Hernes, Espen Olsen, Seth Ayisi Addo, Marit Halonen Christiansen, Arvid Steinar Haugen

**Affiliations:** 1 Faculty of Nursing and Health Science, Nord University, Postboks 1490, Bodø, 8049 Norway; 2 Østfold Hospital Trust, Grålum, Norway; 3 Faculty of Health, Welfare and Organisation, Østfold University College, Halden, Norway; 4 Department of Anaesthesia and Operating Theatres, Nordland Hospital Trust, Bodø, Norway; 5 Sorlandet Hospital Trust HF, Kristiansand, Norway; 6 Department of Clinical Sciences, University of Bergen, Bergen, Norway; 7 Department of Innovation, Management and Marketing, UiS School of Business and Law, University of Stavanger, Stavanger, Norway; 8 Unit of Occupational Medicine, Institute of Environmental Medicine, Karolinska Institutet, Stockholm, Sweden; 9 Department of Obstetrics and Gynaecology, Stavanger University Hospital, Stavanger, Norway; 10 Department of Anaesthesia and Intensive Care, Haukeland University Hospital, Bergen, Norway; 11 Department of Nursing and Health Promotion Acute and Critical Illness, Faculty of Health Sciences, Oslo Metropolitan University, Oslo, Norway

**Keywords:** Hospital Survey on Patient Safety Culture, HSOPSC, Patient safety culture, Operating theatre, Operating room

## Abstract

**Background:**

Patient safety culture encompasses the values, beliefs, norms, and behaviours that influence patient safety. It is positively associated with improved patient outcomes and inversely related to adverse events. This study explores the patient safety culture in the operating theatre setting to support national efforts aimed at mitigating adverse events. Specifically, it assesses the patient safety culture in six Norwegian hospitals and potential associations between scores from the Norwegian Hospital Survey on Patient Safety Culture 2.0 (N-HSOPSC 2.0) scores and socio-demographic factors, including gender, field of expertise, years of experience, leadership responsibilities, and hospital trust.

**Methods:**

An exploratory cross-sectional multicentre study using the N-HSOPSC 2.0 was conducted between 2021 and 2022 among operating theatre staff across six Norwegian hospitals in three hospital trusts. Descriptive statistics were used to report the scores of 10 factors and three outcome variables. Generalised linear models were employed to analyse the relationships between socio-demographic variables (gender, field of expertise, years of experience, leadership responsibilities and hospital trust) and the N-HSOPSC 2.0 scores.

**Results:**

A total of 217 individuals responded to the questionnaire. The teamwork factor had the highest percentage of positive responses (85.8%), while all other factors showed less than 75% positive responses, indicating an unsatisfactory patient safety culture. Notably, three factors had less than 50% positive responses, underscoring the need for targeted improvement initiatives. Respondents with leadership responsibilities reported significantly more positive perceptions of the patient safety culture on three factors and one outcome variable compared to those without such responsibilities. Similarly, individuals identified as unit leaders or administrators reported significantly more positive perceptions of four patient safety culture factors and a more favourable overall patient safety rating. Perceptions varied across different fields of expertise, depending on the specific N-HSOPSC 2.0 factor.

**Conclusion:**

This study underscores the pivotal influence of organisational and societal conditions on the development of patient safety culture in operating theatre settings. It also highlights the necessity for tailored initiatives to address the unique needs of different fields of expertise. Further research is recommended to explore the underlying mechanisms that influence the patient safety culture in operating theatres.

**Supplementary Information:**

The online version contains supplementary material available at 10.1186/s12913-026-14460-y.

## Background

Patient harm is one of the leading causes of disability and death worldwide [1]. On average, one in ten hospital patients is estimated to experience an adverse event [2, 3]. A 2023 study reported that 23.6% of inpatient admissions involved at least one adverse event, 22.7% of which were considered preventable [4]. This challenge has been widely acknowledged since the Institute of Medicine released its landmark report *“To Err Is Human: Building a Safer Health System”*, which highlighted the importance of patient safety culture and proposed measures to improve it [5]. The World Health Organisation also recognises patient safety culture as one of the foundational pillars of patient safety [1]. Despite numerous efforts to enhance patient safety, these issues persist, partly due to the complex and dynamic nature of healthcare systems, which makes it challenging to adapt safety measures from other high-risk organisations [6, 7].

Patient safety culture refers to the collective values, beliefs, norms, and behaviours within an organisation that influence patient safety [8]. This culture can vary across organisational structures, levels, and subcultures [8]. Additionally, organisational culture is shaped by the broader societal culture, which in turn affects the patient safety culture [9]. Bisbey’s patient safety culture framework identifies three subgroups for enabling factors at organisational, group and the individual levels. These factors support the development of a patient safety culture, which in turn influences behaviour and ultimately affects safety outcomes [10]. Research has suggested that cultural differences at a national level also influence the patient safety culture [11]. Several work-related factors have been associated with patient safety culture, including field of expertise [12, 13], leader responsibilities [14], gender [15], years of experience [16], and hospital organisations [2]. A positive workplace culture has been shown to improve patient outcomes and reduce adverse events [17, 18].

The operating theatre is considered one of the hospital settings with the highest prevalence of preventable harm [19]. This heightened risk is attributed to a combination of factors, including surgical and anaesthesia risks, distractions, communication failures, understaffing, human factors, task complexity, and technological challenges [20]. Internationally, a few small observational studies have examined patient safety culture in the operating theatres, revealing variability in patient safety culture across factors such as clinical expertise and levels of training [12, 13]. In Norway, several single-centre and multi-centre studies have investigated patient safety culture in hospitals [21]. However, no multi-centre studies have specifically focused on patient safety culture in Norwegian operating theatre settings.

Exploring factors that influence patient safety culture is critical for ensuring the delivery of safe and high-quality healthcare. This study aims to provide an initial overview of the patient safety culture in six Norwegian operating theatres and to explore potential, hypothesis-generating associations between patient safety culture and socio-demographic factors, including gender, field of expertise, years of experience, leadership responsibility, and hospital trust.

## Method

### Design

An exploratory cross-sectional multicentre design was employed, using the validated Norwegian version of the Hospital Survey on Patient Safety Culture 2.0 (N-HSOPSC 2.0) questionnaire to measure patient safety culture [22]. The study adhered to the Strengthening the Reporting of Observational Studies in Epidemiology statement guidelines for reporting observational studies [23].

### Setting and sample

A purposive sampling strategy was applied to ensure inclusion of hospitals that differed in organisational characteristics such as size, regional role, procedural case-mix and teaching status. The reason for this was to capture contextual variation and differences between fields of expertise rather than statistical representativeness. Healthcare personnel working in operating theatres across six Norwegian hospitals, representing three different hospital trusts, were included to obtain a mix of community and university hospitals as well as coverage of various regions of Norway.

In Norway, the operating team typically comprises nurse anaesthetists, registrar and consultant anaesthetists, operating room nurses, and surgeons. Ancillary personnel provide support services, and unit leaders and administrators oversee the operating theatre personnel. The present study focused on advanced nurse practitioners working in surgical services, physicians responsible for surgical and anaesthesia care, and unit leaders and administrators. The ancillary personnel were excluded, as the study specifically targeted clinical and managerial staff directly involved in patient care and safety practices.

### Data collection

Data collection was conducted between November 2021 and June 2022, using the N-HSOPSC 2.0 instrument, the Norwegian adaptation of the Hospital Survey on Patient Safety Culture 2.0 [24]. This tool has demonstrated acceptable construct validity and reliability in previous research [22]. The N-HSOPSC 2.0 consists of 32 items grouped into ten factors that represent various dimensions of the patient safety culture, along with an open-ended comment section for suggestions to improve patient safety [22, 24]. The factors one to four (“Teamwork”, “Staffing and Work Pace”, “Organisational Learning”, “Response to Error”) and six to eight (”Communication About Error”, “Communication Openness”, “Reporting on Patient Safety Events”) investigate safety culture dimensions at the unit level. Factor five (“Supervisor, Manager or Clinical Leader Support for Patient Safety”) looks at immediate leadership, while the factors nine (“Hospital Management Support for Patient Safety”) and ten (“Handoffs and Information Exchange”) examine safety factors at a hospital level. Factors one to seven, nine and ten use a Likert-like scale where 1 = Strongly Disagree, 2 = Disagree, 3 = Neither Agree nor Disagree, 4 = Agree, 5 = Strongly Agree, and 6 = Does Not Apply or Don’t Know. Factor eight uses a Likert-like scale with 1 = Never, 2 = Rarely, 3 = Sometimes, 4 = Most of the time, 5 = Always, and 6 = Does not Apply or Don’t Know. Two distinct single-item outcome variables are used to represent the underlying construct; how would you rate your unit/work area on patient safety (“Patient Safety Rating”) and how many patient safety events have you reported in the past 12 months (“Number of Events Reported”). “The Patient Safety Rating” is reported using a Likert-like scale where 1 = Poor, 2 = Fair, 3 = Good, 4 = Very Good, and 5 = Excellent. “Number of Events Reported” is reported using a continuous numeric variable.

The N-HSOPSC 2.0 also has an additional outcome variable item on how often the respondent had been present at an adverse event, where he/she didn’t send an incident report (“Adverse Event without Incident Report”). This variable is scored with a Likert-like scale where 1 = Never, 2 = Rarely, 3 = Sometimes, 4 = Often and 5 = Always.

Additional items measured socio-demographic characteristics, including gender, field of expertise (nurse anaesthetist, operating room nurse, anaesthetist, surgeon, unit leader/administrator), years of experience (defined as years in the current speciality), leadership responsibilities (yes/no), and hospital trust(A, B, C).

The questionnaire was distributed to respondents via work e-mails (*N* = 1230), which included a URL link to the survey and an information letter. The respondents completed the questionnaire electronically, and one or two reminders were sent to nonrespondents. The data was collected and securely stored at the University of Oslo’s Service for Sensitive Data (TSD).

### Statistical analysis

Statistical analyses were conducted using IBM SPSS Statistics V. 29.0.2.0 [25]. Negatively worded items and outcome variables were reversed to be aligned with positively scored items. Factor scores were recoded from the Likert-like scale into three categories for descriptive analysis: Negative (≤ 2), Neutral (3), and Positive (≥ 4) responses. Descriptive statistics were used to calculate the percentage of positive responses for each factor [24]. Factors containing ≥ 75% positive responses were defined as patient safety strengths, whereas factors with ≤ 50% positive responses were identified as areas needing improvement [26].

A series of generalised linear models were applied to explore the relationship between independent variables (gender, field of expertise, years of experience, leadership responsibilities, and hospital trust) and the ten patient safety culture factors or the three outcome variables as dependent variables. The sample size was guided by rules of thumb appropriate for exploratory research. In line with Green’s recommended formula for minimum sample size (*N* ≥ 50 + 8k), where k denotes the number of independent variable terms contributing degrees of freedom to the model, the minimum required sample size for this study was 122 participants [27]. The obtained sample size exceeded this threshold. The appropriate link function was selected based on the distribution of the dependent variable and model fit, which was assessed using the Akaike Information Criterion. An identity link function was used for factors one to three, five, seven to nine. In factors four, six, and “Number of Reported Events”, a log link function link was employed. The outcome variables “Patient Safety Rating” and “Adverse Events without Incident Report” were analysed using a cumulative logit link function. The reference categories were selected based on the groups with the greatest differentiation. Prior to the analysis, responses marked as “Does Not Apply or Don’t Know” were recoded into missing values. Missing data were not imputed, and no multiple-testing correction was applied, reflecting the exploratory focus of the analysis.

During data cleaning, it was observed that several factors contained a relatively high proportion of “I don’t know” responses. These responses may reflect uncertainty, limited experience with the specific item content, or limited relevance to the respondent’s work situation [28]. Because selecting this option suggests that the data is “Missing Not At Random”, these were not considered suitable for imputations [29]. Therefore, for factors with more than 10% “I don’t know” responses, Fischer’s exact test was applied to explore whether individuals with and without leadership responsibilities differed in how often they selected this option.

The internal consistency was measured using Cronbach’s alpha. Criterion validity was evaluated through multiple linear regression, with the ten factors as independent variables and the outcome variables as dependent variables. Discriminant validity was examined using Spearman’s Rho correlation. All statistical tests were two-sided, and P-values ≤ 0.05 were considered statistically significant.

The open-ended comments were analysed using a four-step quantitative content analysis process: reading through the comments, coding content categories, recoding into larger recurring categories, and rereading original comments to verify the coding. Each comment could contain references to multiple categories, which were primarily named according to the N-HSOPSC 2.0 factors. The coding process was facilitated using NVivo V. 15.0.0 software [30].

## Results

The data sample consisted of 217 participants from six hospitals: 102 from Hospital Trust A, 102 from Hospital Trust B, and 26 from Hospital Trust C. The approximate response rates were 41.3%, 26.5%, and 6.8%, respectively. Sample characteristics are detailed in Table 1.

As shown in Table 1, the percentage of missing data for gender, field of expertise, years of experience, leadership responsibility and hospital trusts was ≤ 1.8% (*N* ≤ 4). Missing data for the factors and outcome variables of N-HSOPSC 2.0 were ≤ 3.2% (*N* ≤ 7). Detailed item-level missing data are provided in Additional File 1.

**Table 1:** Sample Characteristics (*N* = 217)

Variables	
**Hospital Trusts**, N (%)	
Hospital Trust A	89 (41.0)
Hospital Trust B	102 (47.0)
Hospital Trust C	26 (12.0)
Missing	0 (0.0)
**Gender**, N (%)	
Female	145 (66.8)
Male	71 (32.7)
Missing	1 (0.5)
**Leadership responsibility**, N (%)	
Yes	33 (15.2)
No	183 (84.3)
Missing	1 (0.5)
**Field of Expertise**, N (%)	
Nurse Anaesthetists	77 (35.5)
Operating room nurses	70 (32.3)
Anaesthetists	29 (13.4)
Surgeons	20 (9.2)
Unit Leaders and Administrators	19 (4.1)
Missing	2 (0.9)
**Age**, mean (SD)	48.8 (9.7)
Missing, N (%)	4 (1.8)
**Years of experience**, median (Q1-Q3)	13.59 (5–21)
Missing. N (%)	2 (0.9)

Table 2 shows that in this sample the “Teamwork” factor had 85.8% of the respondents reporting a positive patient safety culture, all other nine factors had < 75% positive responses. The factors “Organisational Learning - Continuous Improvement”, “Reporting on Patient Safety Events”, and “Hospital Management Support for Patient Safety” reported ≤ 50% positive responses. Ordinal and continuous descriptive statistics at the item level are provided in Additional File 1.

**Table 2:** Descriptive statistics of the Hospital Survey on Patient Safety Culture factors (*N* = 217)

Factors	Positive Responses, %	Mean	Std. Deviation	95% CI		Don’t Know Response, *n*	Missing, *n*
				Lower Bound	Upper Bound		
1. Teamwork	85.8	4.22	0.59	4.14	4.3	3	2
2. Staffing and Work Pace	56.1	3.35	0.79	3.24	3.46	10	5
3. Organisational Learning – Continuous Improvement	45.9	3.30	0.79	3.19	3.41	17	3
4. Response to Error	73.3	3.83	0.77	3.71	3.94	29	6
5. Supervisor, Manager or Clinical Leader Support for Patient Safety	71.3	3.86	0.82	3.75	3.98	7	3
6. Communication About Error	69.1	3.69	0.85	3.57	3.81	11	2
7. Communication Openness	72.0	3.76	0.77	3.66	3.87	18	5
8. Reporting on Patient Safety Events	30.4	3.06	0.8	2.94	3.19	57	1
9. Hospital Management Support for Patient Safety	19.3	2.56	0.85	2.44	2.68	22	2
10. Handoffs and Information Exchange	63.1	3.63	0.60	3.54	3.73	49	3
Adverse Events without Incident Report	-	3.75	0.80	3.64	3.86	-	3
Patient Safety Rating	-	3.25	0.83	3.14	3.36	-	2
Number of reported events	-	1.83	2.80	1.45	2.21	-	7

*Abbreviations: 95% CI = 95% confidence interval for the mean.*

**Fig. 1:**
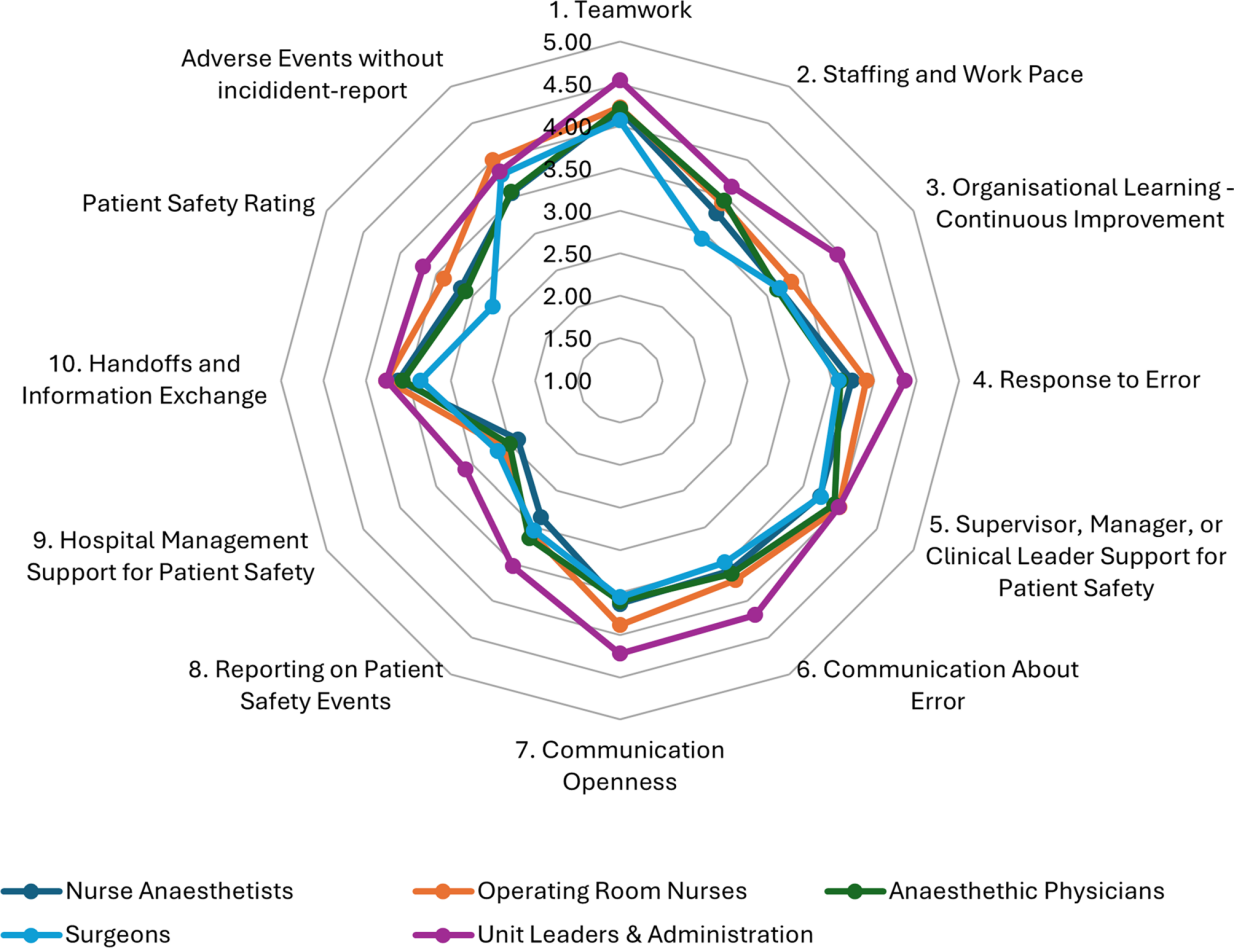
Patient safety culture factors across fields of expertise. Note: Spider diagram illustrating the mean factor score for each field of expertise. The radial axes represent scores from 1–5 on the Likert-type scale used, where 5.0 is the most positive score

Unit leaders and administrators reported the most positive perceptions of safety culture factors and outcomes, except for the outcome variable “Adverse Events without Incident Report”, where, operating room nurses scored higher in our findings. This is illustrated in Fig. 1. The means with corresponding confidence intervals for each field of expertise are provided in Additional File 2.

The generalised linear models suggested significant differences between the field of expertise, with unit leaders and administration, who showed a positive association, serving as the reference category in five out of the ten models. One of these was the patient safety rating, where unit leaders and administrators appeared to have 10 times higher odds of perceiving it positively than surgeons, five times higher odds than anaesthesiologists and 4 times higher odds than nurse anaesthetists (*p* ≤ 0.05). Interestingly, when a statistically significant association was found in “Leadership Responsibility”, no significant association was observed in the “Field of Expertise” under “Unit Leaders and Administrators”. This is likely because the “Leadership Responsibility” variable includes a larger number of respondents in each group compared to the “Field of Expertise”. However, both variables exhibited the same directionality in their significant parameter estimates. Suggesting that leaders, administrators, and those with leadership responsibilities differentiate themselves from the rest in the perception of patient safety culture. Surgeons stood out in the factor “Staffing and Work Pace”, reporting a significantly lower perception than the other fields of expertise. Surgeons also reported on the “Number of Reported Events”, with an incidence rate ratio (IRR) indicating significantly fewer reported events than the other “Fields of Expertise” (*p* ≤ 0.05). This was particularly evident for operating room nurses, who showed the largest difference, with an incidence rate eight times higher than that of the surgeons (IRR = 8.37, *p* ≤ 0.001). A similar pattern appears in the factor “Adverse Events without Incident Report”, where operating room nurses serve as the reference group by having the highest scores. “Years of Experience” was significantly associated with “Response to Error”; for each additional year of experience, the odds of perceiving a more positive “Response to Error” increased by 1%. Indicating that the less experienced may have a more negative perception of how the unit responds to mistakes or event reporting. Hospital Trust C also stood out, showing significant differences in three factors and all outcome variables, while the other two trusts did not differ significantly. However, Hospital Trust C had a notably lower response rate compared to Hospital Trusts A and B. The full generalised linear models can be viewed in Additional File 3.

The response “I don’t know” was more prevalent in two factors: “Reporting on Patient Safety Events” and “Handoffs and Information Exchange”. For “Reporting on Patient Safety Events”, 22.6% of the total responses were “I don’t know”, showing a significant difference between those with leadership responsibility (*p* < 0.001). Among respondents with leadership responsibility, only 3% selected “I Don’t Know”, compared to 30.8% in the group without leadership responsibilities. For the “Handoffs and Information Exchange”, 15.5% of respondents selected “I Don’t Know”. Within the leadership responsibility group, 30% answered, “I Don’t Know”, compared to 21.5% in the non-leadership responsibilities group (*p* = 0.27).

### Reliability and validity measures

The reliability analysis was conducted on the 10 factors of the N-HSOPSC 2.0 questionnaire. The correlation matrix and Cronbach’s alpha coefficients are presented in Table 3. The Cronbach alpha coefficient ranged from 0.47 to 0.86, with only one factor < 0.60.

**Table 3:** Spearman’s Rho and Cronbach’s Alpha

Factor/Factor	1.	2.	3.	4.	5.	6.	7.	8.	9.	10.	11.	12.		13.
1. Teamwork	**0.47**													
2. Staffing and Work Pace	0.18^**^	**0.63**												
3. Organisational Learning – Continuous Improvement	0.35^**^	0.35^**^	**0.69**											
4. Response to Error	0.58^**^	0.45^**^	0.54^**^	**0.79**										
5. Supervisor Support for Patient Safety	0.40^**^	0.36^**^	0.57^**^	0.58^**^	**0.82**									
6. Communication About Error	0.39^**^	0.14	0.61^**^	0.56^**^	0.52^**^	**0.86**								
7. Communication Openness	0.48^**^	0.32^**^	0.58^**^	0.63^**^	0.61^**^	0.56^**^	**0.81**							
8. Reporting on Patient Safety Events	0.36^**^	0.28^**^	0.45^**^	0.46^**^	0.38^**^	0.51^**^	0.39^**^	**0.74**						
9. Hospital Management Support for Patient Safety	0.25^**^	0.34^**^	0.40^**^	0.26^**^	0.29^**^	0.38^**^	0.34^**^	0.21^**^	**0.78**					
10. Handoffs and Information Exchange	0.23^**^	0.15	0.23^**^	0.37^**^	0.32^**^	0.23^**^	0.15	0.11	0.12	**0.60**				
11. Adverse Events without Incident Report	0.09	0.14^*^	0.31^**^	0.22^**^	0.31^**^	0.23^**^	0.24^**^	0.23^**^	0.19^**^	0.06	**-**			
12. Patient Safety Rating	0.39^**^	0.35^**^	0.48^**^	0.49^**^	0.53^**^	0.34^**^	0.49^**^	0.48^**^	0.29^**^	0.41^**^	0.31^**^	**-**		
13. Number of Reported Events	0.05	− 0.14	− 0.01	0.00	0.02	0.11	0.10	− 0.01	− 0.16^*^	− 0.04	− 0.19^**^	0.07		**-**

**. Correlation is significant at the 0.01 level (2-tailed).

*. Correlation is significant at the 0.05 level (2-tailed).

Abbreviations: (1) Teamwork, (2) Staffing and Work Pace, (3) Organisational Learning - Continuous Improvement, 5. Supervisor, Manager, or Clinical Leader Support for Patient Safety, 7. Communication Openness, 8. Reporting on Patient Safety Events, 9. Hospital Management Support for Patient Safety, 10. Handoffs and Information Exchange, (4) Response to Error, 6. Communication About Error, 11. Adverse Events without Incident Report, 12. Patient Safety Rating, 13. Number of reported events

The correlations between factors were low enough to suggest that the factors measure distinct constructs, thereby supporting discriminant validity. For example, “Number of Reported Events” significantly correlated only with “Hospital Management Support for Patient Safety” (r(186) = 0.189, *p* < 0.005). In contrast, “The Adverse Events without Incident Report” did not significantly correlate with “Teamwork” (r(208) = 0.123, *p* = 0.075) or “Staffing and Work Pace” (r(198) = 0.107, *p* = 0.132). Significant correlations that were observed had *r* ≤ 0.312, further supporting the distinctiveness of the factors.

The “Patient Safety Rating” significantly correlated with all the factors (*r* > 0.294, *p* < 0.010), indicating that it reflects an underlying construct related to overall patient safety.

Criterion validity was assessed using multiple linear regression. For the “Patient Safety Rating”, the model explained 50.8% of the variance (R^2^ = 0.508). In comparison, “Number of Reported Events” explained 10.5% of the variance (R^2^ = 0.105), and “Adverse Events without Incident Report” explained 13.2% of the variance (R^2^ = 0.132).

## Open-ended responses

A total of 66 out of 217 respondents provided input in the open-ended comment section, sharing insights on how current practices or potential changes might influence patient safety. Through a quantitative content analysis, 110 references were identified and grouped into eight categories. The four categories labelled “Reporting on Errors”, “Staffing”, “Continuous Learning”, and “Openness About Errors” were named to reflect their close alignment with the corresponding factors in the N-HSOPSC 2.0. The remaining four diverge from the established factor structure and provide additional insights into safety work in the operating theatre context. The most frequent mentioned categories were “Staffing”, “Quality vs. Production”, “Continuous Learning” and “Cooperation”. An overview of these categories is presented in Table 4.

**Table 4:** Quantitative content analysis of the open-ended comments (*N* = 66)

Category	Description	Frequency	%
Reporting Errors	Described as time-consuming, with no feedback provided on reported events.	7	6
Staffing	Compromises patient safety in general and during shifts due to productivity demands.	30	27
Continuous Learning	Focuses on time for simulation, staying updated professionally, and learning from past adverse events.	15	14
Quality vs. Production	Production priorities overshadow time spent familiarizing with patient cases.	20	18
Leadership	Lacking focus on patient safety, worsening at higher leadership levels, with poor follow-up on safety routines.	6	5
Patient Safety-Promoting Structures	Highlights the need for more routines, procedures, and standards to ensure safety.	4	4
Cooperation	Encompasses interdisciplinary and intradisciplinary collaboration, advocating for regular safety meetings and improved team communication.	15	14
Openness About Errors	Stresses the importance of easily expressing safety concerns and focusing on reporting utility, not fear.	13	12

Note: Frequency in this table represent the total number of times a category is mentioned across all respondents

## Discussion

This is the first study exploring patient safety culture across various Norwegian operating theatres. The results suggest that “Teamwork” was the only patient safety culture factor identified as a strength, whereas “Organisational Learning”, “Reporting on Patient Safety Events” and “Hospital Management Support for Patient Safety” were recognised as weaknesses. Furthermore, field of expertise, years of experience and leadership responsibility appeared to be associated with patient safety culture among operating theatre personnel. The high proportion of “I don’t know” responses within the factor “Reporting on Patient Safety Events” suggests that those with leadership responsibilities were better positioned to assess this domain. These findings suggest that patient safety culture is insufficient in key areas that are strongly influenced by leadership and organisational practices.

### Overarching strengths and weaknesses in patient safety culture

The identification of “Teamwork” as a strength aligns with prior findings from both international and national studies using HSOPSC data [31–37]. However, “Organisational Learning” was identified as a weakness in this study, diverging from international findings, where it is often reported as a strength [31, 32]. This weakness is consistent with earlier Norwegian studies, which have similarly found a weak culture in “Organisational learning” [33–36, 38]. Interestingly, our results for “Response to Error” indicate a highly positive perception, which is consistent with previous Norwegian studies [33–36, 38], and contrasts with international findings where this factor is typically rated low [31, 32]. This discrepancy may reflect characteristics of Norwegian societal culture, particularly low power distance and egalitarian norms, that foster expectations of fair and non-punitive responses to error. Organisations often mirror these broader societal norms [39, 40]. Similarly, “Reporting on Patient Safety Events” was perceived as low in this study, reflecting both national and international trends [31, 33–36, 38]. Low ratings for “Hospital Management Support for Patient Safety” also align with earlier research [31–34, 36], although newer research suggests improvement in this area [35]. Differences between national and international findings may therefore reflect broader societal culture influences rather than organisational variations across healthcare organisations [9]. Evidence suggests that meaningful variation in safety culture within a country occurs primarily at the clinical or unit level, whereas some variation tends to be associated with the hospital level [11]. At the organisational level, differences between hospital trusts were observed in the factors “Staffing and Work Pace” and “Hospital Management Support for Patient Safety”. Differences were also identified in the three outcome variables. Significant findings were detected only when compared with Hospital Trust C, the only university hospital in our sample. Similar variations in the perceptions of factors influencing the operating theatre safety culture have been reported between university and county hospitals in Sweden [16]. From the perspective of Bisbey’s patient safety culture framework, these weaknesses reflect gaps in organisational enabling conditions, indicating that system-level processes, rather than team-level dynamics, largely influence frontline staff’s perceptions [10]. This interpretation is supported by the findings from the open-ended comments, which consistently referred to strategic management of patient safety and learning. The weaknesses in “Hospital Management Support for Patient Safety” and “Continuous Learning” have also been identified in previous Norwegian research in the operating theatre setting context [38]. Structural equation modelling has shown that “Continuous Learning” forms a key pathway between “Hospital Management Support for Patient Safety” and patient safety rating in Norwegian hospitals [41]. Taken together, this pattern indicates that the way patient safety is operationalised and supported by unit leaders and administrators is not perceived as adequate by frontline staff. It is well established that thoroughly addressing past patient safety issues can contribute to improving future efficiency [7].

### Patient safety culture across fields of expertise

Perceptions of patient safety culture varied across fields of expertise. Comparable studies from the US operating setting have shown that surgeons hold the most positive perception of patient safety culture, although these studies did not include unit leaders and administrators [12, 13]. Conversely, earlier Norwegian research has found that anaesthetic personnel report the most positive perceptions [38]. In the present study, unit leaders and administrators in fields of expertise rated patient safety culture highest across 12 out of 13 factors and outcome variables. This finding is consistent with previous findings showing that hospital leadership tend to report more positively in safety and quality assessments [14]. Earlier research suggests that middle managers identify limited resources as a key barrier to supporting patient-safety initiatives at the frontline [42]. However, this does not explain the perceptual gap between frontline staff and leadership. Notably, those with leadership responsibilities appear to be more than twice as likely to report adverse events, suggesting a greater exposure to safety-related administrative processes. Our findings picture an information and resource asymmetry between leadership and frontline staff. This could either reflect an actual, more robust safety culture within leadership levels, or it may stem from unit leaders and administrators holding an overly optimistic view due to variations in the information they receive or how incidents are reported. Understanding these dynamics is critical for leaders to reconcile “work-as-done” with “work-as-imagined” and to help healthcare staff develop the knowledge required to effectively identify potential risks and errors [7].

The quantitative content analysis further reinforces this interpretation, comments regarding staffing, continuous learning, and quality vs. production collectively point to insufficient resources or prioritisation for safety work. Additionally, the high prevalence of “I don’t know” responses suggests that leadership responsibility plays a role in staff’s ability to assess the “Reporting on Patient Safety Events” factor. Importantly, “I don’t know” responses should not be treated as missing but may reflect a missing-not-at-random mechanism [43]. This type of analysis has not been conducted previously, as the earlier versions of the HSOPSC questionnaire did not include this response option. This may indicate limited knowledge among those without leadership responsibilities about previous safety events. Contrary to expectations that managers promote patient safety through systematic event reporting, and frontline staff discussing patient safety [44].

Surgeons expressed concerns about staffing levels and work pace, reporting significantly lower perceptions for “Staffing and Work Pace” than for other fields of expertise. This finding is consistent with other studies that reported differences in the perception of “Staffing and Work Pace” between surgeons and nurses [45]. Surgeons were also less likely to report patient safety incidents, suggesting potential cultural barriers to reporting safety concerns. In contrast, operating room nurses displayed the highest number of reported adverse events. In Bisbey’s framework, one enabling factor for patient safety culture is group-level psychological safety, defined as the group being “safe for interpersonal risk taking” [10]. Surgeons’ hesitation to report may stem from concerns about self-incriminating or reputational consequences, given that reporting may implicitly identify them as a risk factor [46]. The open-ended comments do not directly correspond to the field of expertise, but offer an alternative explanation, a lack of feedback may limit reporting behaviour. However, they also highlight concerns about insufficient openness regarding errors. These dynamics likely contribute to surgeons’ lower overall safety ratings among the fields of expertise.

Years of experience appeared to be significantly associated with an increase in the factor “Response to Error”. This finding aligns with other studies from Sweden, where lower younger age and fewer years of experience have been associated with lower perceptions of patient safety culture factors [16, 47]. Within Bisbey’s framework, years of experience represent an individual-level attribute, shaping staff’s personal perception of safety-related processes [10]. Over time, experiences with speaking up can foster improved perceptions of expressing concerns and greater familiarity within teams [48]. This is particularly relevant in the operating theatre setting, where hierarchical structures are more pronounced and interdisciplinary collaborations are essential for positive patient outcomes [49].

Overall, the patterns identified in this discussion point toward a potential mismatch between how organisational leadership operationalises, communicates and supports safety, and how these efforts are perceived by frontline staff. While these interpretations should be viewed in the light of the exploratory nature of the study, they align with established literature on psychological safety, which links higher levels of psychological safety to information sharing, positive leader relationships, and organisational learning [50]. These preliminary connections provide a basis for developing hypotheses and identifying areas warranting further empirical investigation.

### Strengths and limitations

A key strength of this study is its exploration of patient safety culture across multiple professional groups within the same clinical context, and comparison within a shared societal culture [9]. A methodological strength of this study is its inclusion of six hospitals across three different hospital trusts, encompassing both university and community hospitals. This increases organisational heterogeneity and allows exploration of patient safety culture across different institutional contexts, thereby strengthening the analytical generalisability of the findings within the Norwegian operating theatre setting.

At the same time, relatively low and uneven response rates, particularly from hospital Trust C, limit statistical representativeness and may reduce external validity. Thus, while the multicentre design enhances the contextual breadth, the modest participation rate constrains the extent to which the findings can be generalised to all operating theatres nationally.

The response pattern from Hospital Trust C has been observed in later data collections, suggesting a possible survey response fatigue. Moreover, there was a notable increase in survey activity during the COVID-19 pandemic, which has been reported to contribute to declining overall response rates [51]. Low response rates have also been reported in other surveys on patient safety culture conducted during the COVID-19 pandemic [52].

The varying response rates across fields of expertise and hospital trusts may introduce both response bias and non-response bias. Consequently, the descriptive analyses are particularly vulnerable to non-response bias and should be interpreted with caution. Although the generalised linear models were adjusted for relevant covariates, statistical adjustment cannot compensate for potential selection mechanisms in the sample.

Additionally, the Cronbach’s alpha for the “Teamwork” factor was less than 0.60, which may have been influenced by a negatively worded item (see Additional File 1). Comparisons between studies should also be interpreted cautiously because two different versions of the HSOPSC tool were used [24]. Finally, as this was an exploratory study, no correction for multiple testing was applied in the univariate regression analysis. This increases the risk of a type I error, and the results should therefore be interpreted with caution.

## Conclusion

This exploratory study highlights the pivotal influence of organisational and societal conditions on the development of patient safety culture, underscoring the different challenges across different fields of expertise and the need for tailored initiatives. The findings demonstrate the importance of conducting systematic assessments of the patient safety culture to address existing gaps.

The results suggest that those with leadership responsibilities may have greater access to information and are more frequently exposed to patient safety work, thereby strengthening their perception that safety efforts are well established. However, these initiatives may not always effectively reach frontline staff. Limited upward reporting from frontline personnel may also contribute to unit leaders and administrators holding an overly optimistic view of patient safety. This disconnect between leadership and frontline staff may hinder the development of a robust patient safety culture.

Further research is needed to explore the underlying mechanisms linking the work environment, occupational health, and patient safety culture in operating theatres. Understanding how patient safety culture may function as a mediating mechanism can provide valuable insights into the processes that shape safety outcomes, thereby informing the development of more effective strategies to strengthen culture for both healthcare providers and the patients they serve.

## Supplementary Information

Below is the link to the electronic supplementary material.

Supplementary Material 1

Supplementary Material 2

Supplementary Material 3

## Data Availability

The datasets analysed during the current study are not publicly available due to local ownership policies. However, aggregated data can be obtained from the corresponding author on reasonable request.

## Abbreviations

N-HSOPSC 2.0: Hospital Survey on Patient Safety Culture 2.0
TSD: University of Oslo’s Service for Sensitive Data
