# Supplementary material for: Exploring demographic and organisational variations in patient safety culture: a cross-sectional, multicentre study in operating theatres of six Norwegian hospitals
Source: BMC Health Serv Res. 2026 Mar 31;26:678. doi: 10.1186/s12913-026-14460-y (PMC13162434; doi:10.1186/s12913-026-14460-y)
Supplement: Supplementary file 1 — Supplementary Material 1 [file 12913_2026_14460_MOESM1_ESM.docx]

| **Additional File 2** Patient safety culture factors and outcome according to field of expertise (N = 217) | | | | | | | | | |  |
| --- | --- | --- | --- | --- | --- | --- | --- | --- | --- | --- |
| **Patient Safety Culture Factors** | **Anaesthethic Physicians, N = 29** | | **Nurse Anaesthetists,**  **N = 77** | | **Surgeons, N = 20** | | **Operating Room Nurses, N = 70** | | **Leaders and Administrators, N = 19** | |
|  | Mean(SD) | 95% CI | Mean(SD) | 95% CI | Mean(SD) | 95% CI | Mean(SD) | 95% CI | Mean(SD) | 95% CI |
| **1.** | 4.21 (.78) | 3.91 - 4.51 | 4.19 (.55) | 4.06 - 4.31 | 4.10 (.57) | 3.84 - 4.35 | 4.22 (.58) | 4.08 - 4.36 | 4.54 (.40) | 4.35 - 4.74 |
| **2.** | 3.45 (.90) | 3.09 - 3.81 | 3.28 (.72) | 3.11 - 3.44 | 3.03 (.94) | 2.57 - 3.48 | 3.41 (.77) | 3.22 - 3.60 | 3.64 (.71) | 3.28 - 3.99 |
| **3.** | 3.14 (.91) | 2.77 - 3.51 | 3.17 (.75) | 2.98 - 3.35 | 3.27 (.69) | 2.96 - 3.58 | 3.33 (.76) | 3.14 - 3.52 | 3.96 (.65) | 3.64 - 4.28 |
| **4.** | 3.60 (1.06) | 3.18 - 4.02 | 3.73 (.76) | 3.54 - 3.93 | 3.68 (.67) | 3.33 - 4.02 | 3.91 (.65) | 3.74 - 4.08 | 4.36 (.51) | 4.11 - 4.61 |
| **5.** | 3.91 (.97) | 3.55 - 4.29 | 3.73 (.85) | 3.53 - 3.92 | 3.79 (.77) | 3.44 - 4.14 | 3.98 (.73) | 3.80 - 4.17 | 3.98 (.81) | 3.58 - 4.39 |
| **6.** | 3.63 (1.02) | 3.24 - 4.02 | 3.59 (.81) | 3.40 - 3.78 | 3.54 (1.05) | 3.06 - 4.02 | 3.72 (.73) | 3.54 - 3.90 | 4.19 (.75) | 3.83 - 4.55 |
| **.7** | 3.60 (1.07) | 3.18 - 4.03 | 3.63 (.78) | 3.44 - 3.82 | 3.59 (.69) | 3.23 - 3.96 | 3.88 (.63) | 3.73 - 4.04 | 4.22 (.45) | 4.00 - 4.45 |
| **.8** | 3.15 (.89) | 2.77 - 3.52 | 2.86 (.74) | 2.67 - 3.06 | 3.11 (.71) | 2.70 - 3.52 | 3.08 (.85) | 2.82 - 3.34 | 3.53 (.61) | 3.27 - 3.83 |
| **.9** | 2.51 (1.03) | 2.10 - 2.91 | 2.39 (.76) | 2.21 - 2.58 | 2.67 (.72) | 2.30 - 3.04 | 2.58 (.90) | 2.35 - 2.82 | 3.11 (.69) | 2.77 - 3.44 |
| **.10** | 3.56 (.65) | 3.30 - 3.83 | 3.61 (.55) | 3.48 - 3.75 | 3.39 (.64) | 3.06 - 3.72 | 3.76 (.59) | 3.58 - 3.93 | 3.76 (.75) | 3.26 - 4.26 |
| **.11** | 3.57 (.84) | 3.25 - 3.90 | 3.55 (.77) | 3.38 - 3.73 | 3.82 (.96) | 3.39 - 4.24 | 4.00 (.73) | 3.83 - 4.17 | 3.84 (.69) | 3.51 - 4.17 |
| **.12** | 3.1 (.86) | 2.78 - 3.43 | 3.17 (.79) | 2.99 - 3.35 | 2.81 (.87) | 2.41 - 3.21 | 3.40 (.81) | 3.21 - 3.59 | 3.68 (.67) | 3.36 - 4.01 |
| **.13** | 1.11 (1.3) | 0.61 - 1.61 | 1.95 (2.65) | 1.34 - 2.56 | 0.24 (.63) | 0.00 - 0.52 | 2.32 (3.55) | 1.46 - 3.18 | 2.44 (2.77) | 1.07 - 3.82 |
| *Abbrevations: 1. Teamwork, 2. Staffing and Work Pace, 3. Organisational Learning - Continuous Improvement, 4. Response to Error, 5. Supervisor, Manager, or Clinical Leader Support for Patient Safety, 6. Communication About Error, 7. Communication Openness, 8. Reporting on Patient Safety Events, 9. Hospital Management Support for Patient Safety, 10. Handoffs and Information Exchange, 11. Adverse Events without incident-report, 12. Patient Safety Rating, 13. Number of reported events, SD = Standard deviation, 95% CI = 95% Confidence Intervals. Note: Number of reported events is count data.* | | | | | | | | | | |
