# Supplementary material for: Exploring demographic and organisational variations in patient safety culture: a cross-sectional, multicentre study in operating theatres of six Norwegian hospitals
Source: BMC Health Serv Res. 2026 Mar 31;26:678. doi: 10.1186/s12913-026-14460-y (PMC13162434; doi:10.1186/s12913-026-14460-y)
Supplement: Supplementary file 2 — Supplementary Material 2 [file 12913_2026_14460_MOESM2_ESM.docx]

| **Additional file 1** Descriptive statistics on item and factor level | | | | | | | | | | |
| --- | --- | --- | --- | --- | --- | --- | --- | --- | --- | --- |
| **Factor** | **Items** | **Response** | | | | |  |  | **95% CI** | |
|  |  | Negative | Neutral | Positive | Don’t Know | Missing | Mean | Std. Deviation | Lower Bound | Upper Bound |
| 1. Teamwork | | 5.53% | 8.45% | 84.79% | 0.61% | 0.61% | 4.22 | 0.59 | 4.14 | 4.30 |
|  | In this unit, we work together as an effective team. | 3.69% | 7.83% | 87.10% | 0.92% | 0.46% | 4.38 | 0.82 | 4.27 | 4.49 |
|  | During busy times, staff in this unit help each other. | 0.46% | 3.69% | 94.01% | 0.92% | 0.92% | 4.41 | 0.59 | 4.33 | 4.49 |
|  | There is a problem with disrespectful behaviour by those working in this unit. | 12.44% | 13.82% | 73.27% | 0.00% | 0.46% | 3.87 | 1.09 | 3.72 | 4.01 |
| 2. Staffing and Work Pace | | 26.38% | 16.47% | 54.84% | 1.38% | 0.92% | 3.35 | 0.79 | 3.24 | 3.46 |
|  | In this unit, we have enough staff to handle the workload | 36.87% | 11.98% | 49.77% | 0.00% | 1.38% | 3.19 | 1.18 | 3.03 | 3.35 |
|  | Staff in this unit work longer hours than is best for patient care. | 11.52% | 13.36% | 72.35% | 1.84% | 0.92% | 3.83 | 1.05 | 3.69 | 3.97 |
|  | This unit relies too much on temporary, float, or PRN staff. | 27.65% | 13.36% | 55.30% | 3.23% | 0.46% | 3.32 | 1.30 | 3.14 | 3.50 |
|  | The work pace in this unit is so rushed that it negatively affects patient safety. | 29.49% | 27.19% | 41.94% | 0.46% | 0.92% | 3.07 | 1.06 | 2.93 | 3.21 |
| 3. Organisational Learning - Continuous Improvement | | 19.82% | 32.10% | 44.09% | 3.23% | 0.77% | 3.30 | 0.79 | 3.19 | 3.41 |
|  | This unit regularly reviews work processes to determine if changes are needed to improve patient safety. | 20.74% | 28.57% | 48.85% | 1.38% | 0.46% | 3.34 | 1.06 | 3.20 | 3.49 |
|  | In this unit, changes to improve patient safety are evaluated to see how well they worked. | 20.74% | 36.87% | 37.33% | 4.15% | 0.92% | 3.21 | 0.97 | 3.08 | 3.35 |
|  | This unit lets the same patient safety problems keep happening. | 17.97% | 30.88% | 46.08% | 4.15% | 0.92% | 3.35 | 0.97 | 3.22 | 3.49 |
| 4. Response to Error | | 10.83% | 14.17% | 68.78% | 5.18% | 1.04% | 3.83 | 0.77 | 3.71 | 3.94 |
|  | In this unit, staff feel like their mistakes are held against them. | 7.83% | 8.76% | 78.80% | 3.69% | 0.92% | 4.06 | 0.97 | 3.93 | 4.20 |
|  | When an event is reported in this unit, it feels like the person is being written up, not the problem. | 9.68% | 11.06% | 74.19% | 3.69% | 1.38% | 3.96 | 0.99 | 3.82 | 4.10 |
|  | When staff make errors, this unit focuses on learning rather than blaming individuals. | 5.53% | 11.52% | 80.65% | 1.84% | 0.46% | 4.09 | 0.86 | 3.97 | 4.21 |
|  | In this unit, there is a lack of support for staff involved in patient safety errors. | 20.28% | 25.35% | 41.47% | 11.52% | 1.38% | 3.26 | 1.07 | 3.11 | 3.41 |
| 5. Supervisor, Manager, or Clinical Leader Support for Patient Safety | | 9.06% | 18.89% | 69.59% | 1.69% | 0.77% | 3.86 | 0.82 | 3.75 | 3.98 |
|  | My supervisor, manager, or clinical leader seriously considers staff suggestions for improving patient safety. | 6.45% | 12.90% | 77.88% | 1.84% | 0.92% | 4.06 | 0.93 | 3.93 | 4.18 |
|  | My supervisor, manager, or clinical leader wants us to work faster during busy times, even if it means taking shortcuts. | 14.29% | 21.20% | 62.67% | 0.92% | 0.92% | 3.68 | 1.07 | 3.53 | 3.82 |
|  | My supervisor, manager, or clinical leader takes action to address patient safety concerns that are brought to their attention. | 6.45% | 22.58% | 68.20% | 2.30% | 0.46% | 3.84 | 0.88 | 3.72 | 3.96 |
| 6. Communication About Error | | 13.36% | 16.74% | 67.28% | 2.00% | 0.61% | 3.69 | 0.85 | 3.57 | 3.81 |
|  | We are informed about errors that happen in this unit. | 17.05% | 17.97% | 62.67% | 1.84% | 0.46% | 3.56 | 1.02 | 3.42 | 3.69 |
|  | When errors happen in this unit, we discuss ways to prevent them from happening again | 11.98% | 16.13% | 70.05% | 1.38% | 0.92% | 3.77 | 0.94 | 3.64 | 3.89 |
|  | In this unit, we are informed about changes that are made based on event reports. | 11.06% | 16.13% | 69.12% | 2.76% | 0.92% | 3.73 | 0.92 | 3.60 | 3.85 |
| 7. Communication Openness | | 10.25% | 16.71% | 69.47% | 2.65% | 0.92% | 3.76 | 0.77 | 3.66 | 3.87 |
|  | In this unit, staff speak up if they see something that may negatively affect patient care | 7.37% | 7.83% | 83.41% | 0.46% | 0.92% | 3.98 | 0.86 | 3.87 | 4.10 |
|  | When staff in this unit see someone with more authority doing something unsafe for patients, they speak up. | 11.06% | 22.12% | 58.06% | 7.37% | 1.38% | 3.63 | 0.93 | 3.50 | 3.76 |
|  | When staff in this unit speak up, those with more authority are open to their patient safety concerns | 12.44% | 22.58% | 62.21% | 2.30% | 0.46% | 3.63 | 0.98 | 3.50 | 3.76 |
|  | In this unit, staff are afraid to ask questions when something does not seem right. | 10.14% | 14.29% | 74.19% | 0.46% | 0.92% | 3.86 | 0.99 | 3.73 | 3.99 |
| 8. Reporting on Patient Safety Events | | 20.05% | 33.41% | 23.50% | 22.58% | 0.46% | 3.06 | 0.80 | 2.94 | 3.19 |
|  | When a mistake is caught and corrected before reaching the patient, how often is this reported? | 19.35% | 36.41% | 21.66% | 22.12% | 0.46% | 3.02 | 0.88 | 2.89 | 3.16 |
|  | When a mistake reaches the patient and could have harmed the patient, but did not, how often is this reported? | 20.74% | 30.41% | 25.35% | 23.04% | 0.46% | 3.11 | 0.91 | 2.97 | 3.25 |
| 9. Hospital Management Support for Patient Safety | | 44.85% | 30.72% | 18.13% | 5.68% | 0.61% | 2.56 | 0.85 | 2.44 | 2.68 |
|  | The actions of hospital management show that patient safety is a top priority. | 35.94% | 30.88% | 28.11% | 4.61% | 0.46% | 2.83 | 1.10 | 2.68 | 2.98 |
|  | Hospital management provides adequate resources to improve patient safety | 52.53% | 29.49% | 10.14% | 6.91% | 0.92% | 2.37 | 0.95 | 2.23 | 2.50 |
|  | Hospital management seems interested in patient safety only after an adverse event happens. | 46.08% | 31.80% | 16.13% | 5.53% | 0.46% | 2.51 | 0.99 | 2.38 | 2.65 |
| 10. Handoffs and Information Exchange | | 7.53% | 23.35% | 52.84% | 15.51% | 0.77% | 3.63 | 0.60 | 3.54 | 3.73 |
|  | When transferring patients from one unit to another, important information is often left out. | 12.90% | 32.26% | 42.86% | 11.06% | 0.92% | 3.37 | 0.87 | 3.25 | 3.50 |
|  | During shift changes, important patient care information is often left out. | 3.69% | 20.28% | 56.22% | 19.35% | 0.46% | 3.78 | 0.77 | 3.67 | 3.90 |
|  | During shift changes, there is adequate time to exchange all key patient care information. | 5.99% | 17.51% | 59.45% | 16.13% | 0.92% | 3.77 | 0.78 | 3.66 | 3.89 |
| *Note: Continuous Results are calculated with pairwise exclusion of cases. Response columns are counted, then presented in percentages.* | | | | | | | | | | |
