# Supplementary material for: Exploring demographic and organisational variations in patient safety culture: a cross-sectional, multicentre study in operating theatres of six Norwegian hospitals
Source: BMC Health Serv Res. 2026 Mar 31;26:678. doi: 10.1186/s12913-026-14460-y (PMC13162434; doi:10.1186/s12913-026-14460-y)
Supplement: Supplementary file 3 — Supplementary Material 3 [file 12913_2026_14460_MOESM3_ESM.docx]

| **Additional File 3** Associations between Patient Safety Culture, Field of Expertise, Hospital Trust and Sociodemographic factors (N=217). | | | | | | | | | | | | | | |
| --- | --- | --- | --- | --- | --- | --- | --- | --- | --- | --- | --- | --- | --- | --- |
| **Independent Variable/Dependent Variable** | | **1.** | **2.** | **3.** | **5.** | **7.** | **8.** | **9.** | **10.** | **4.** | **6.** | **11.** | **12.** | **13.** |
|  |  | B Coefficient | | | | | | | | OR | | | | IRR |
| **Field of Expertise** | Nurse Anaesthetists | -.39* | .65* | -.63* | -.28* | -.33 | -.61* | -.95*** | .13 | .93 | 1.19 | .31*** | .25* | 6.80*** |
|  | Operating Room Nurses | -.35 | .86*** | -.44 | 0 | -.06 | -.36 | -.71* | .26 | .85* | 1.14 | 1 | .50 | 8.37*** |
|  | Anaesthesiologists | -.39 | .71** | -.70* | -.23 | -.45 | -.45 | -.92** | 0 | 1 | 1.18 | .23** | .19* | 3.76* |
|  | Surgeons | -.43* | 0 | -.60* | -.33 | -.47 | -.35 | -.72* | .01 | .96 | 1.17 | .39 | .10** | 1 |
|  | Unit Leaders | 0 | .90** | 0 | -.20 | 0 | 0 | 0 | .26 | .81 | 1 | .50 | 1 | 5.40*** |
| **Hospital Trust** | Hospital Trust A | 0 | 0 | .17 | .35 | -.14 | -.18 | -.64*** | 0 | 1.06 | .89 | 2.74* | 2.61* | 1 |
|  | Hospital Trust B | -.03 | .33 | .15 | .20 | -.17 | -.24 | -.49* | -.24 | 1.07 | 1 | 3.71** | 1.85 | .92 |
|  | Hospital Trust C | -.12 | .45*** | 0 | 0 | 0 | 0 | 0 | -.25* | 1 | .96 | 1 | 1 | .76* |
| **Gender**, male | | .03 | .19 | .06 | .27 | .09 | .10 | .10 | .19 | .99 | 1.02 | 1.22 | 1.56 | .99 |
| **Leadership Responsibility**, yes | | .04 | -.04 | .23 | .26 | .43* | .17 | -.29 | .08 | .78** | .83* | 1.07 | 1.35 | 2.13*** |
| **Years of Experience** | | .00 | .01 | .00 | .00 | .00 | .00 | .01 | .00 | .99* | 1.00 | 1.01 | 1.00 | 1.00 |
| **AIC** | | 384 | 458 | 451 | 494 | 434 | 374 | 469 | 296 | 366 | 469 | 437 | 456 | 910 |
| **Missing**, N | | 10 | 20 | 25 | 16 | 28 | 61 | 29 | 55 | 39 | 18 | 8 | 7 | 13 |
| ** ≤ 0.05, ** ≤ 0.005, *** ≤ 0.001 significance probability. Note: Generalised Linear Models were used with the appropriate link function for each HOSPSC factor and outcome variable. The Likert-like scale in factors 4 and 6 is inverted to fit the gamma distribution. Adverse Events without incident reports and Patient Safety are using ordinal rank. Unit Leaders include Administrators. Missing may also be due to the “Don’t Know” response.  Abbreviations: 1. Teamwork, 2. Staffing and Work Pace, 3. Organisational Learning - Continuous Improvement, 5. Supervisor, Manager, or Clinical Leader Support for Patient Safety, 7. Communication Openness, 8. Reporting on Patient Safety Events, 9. Hospital Management Support for Patient Safety, 10. Handoffs and Information Exchange, 4. Response to Error, 6. Communication About Error, 11. Adverse Events without Incident Report, 12. Patient Safety Rating, 13. Number of reported events, AIC = Akaike Information Criterion, OR = Odds Ratio, IRR = Incidence Rate Ratio* | | | | | | | | | | | | | | |
